# Mechanisms of drowning in children: influence of cold shock response on repolarization patterns and arrhythmia burden in healthy children

**DOI:** 10.3389/fspor.2026.1809042

**Published:** 2026-07-09

**Authors:** S. Peter, A. Michaelis, R. Wagner, R. P. Marshall, M. Bovet, J. Weickmann, M. Weidenbach, I. Dähnert, C. Paech

**Affiliations:** 1 Department for Pediatric Cardiology, University of Leipzig - Heart Center, Leipzig, Germany; 2 Pediatric Practice, Leipzig, Germany; 3 RasenBallsport Leipzig GmbH, Leipzig, Germany; 4 Department of Orthopedic and Trauma Surgery, Martin-Luther-University Halle-Wittenberg, Halle, Germany

**Keywords:** arrhythmia, children, cold shock response, drowning, repolarization, swimming

## Abstract

**Introduction:**

While drowning plays a particularly important role in children, the data on the physiology of drowning in this population are scarce. Both the cold shock response and the development of arrhythmias within the context of an autonomic conflict may be of particular significance during drowning. The cold shock response has now been outlined by our working group, while the effect of cold water on cardiac repolarization remains unclear. This study aims to provide a more detailed examination of the effects of cold shock response in healthy children, focusing on changes in repolarization patterns and the development of arrhythmias to enhance the knowledge on drowning mechanisms in children.

**Methods:**

Participants were first immersed up to the neck in warm water (34 °C) and then in cold water (11 °C), while skin temperature, heart rate and respiratory rate were continuously measured and ECG and Holter-ECG were recorded.

**Results:**

Heart rate variability parameters were lower in cold water compared to warm water. In cold water, the Tp-Te interval was significantly shorter compared to baseline in air. Additionally, QT interval did not adequately adapt to the sudden increase in heart rate during cold water immersion. Despite premature contractions regardless of immersion, no arrhythmias were detected.

**Conclusion:**

The current study presents first exploratory data on repolarization patterns and arrhythmia burden in healthy children during the immersion into 11 °C (52°F) cold water. Data suggest, that cold water immersion represents a strong sympathetic stressor and may be associated with a mild QT hysteresis and a distinct influence on the transmural gradient of repolarization. Although no arrhythmias were detected during cold water immersion in healthy children, the cold shock response itself appears to have some influence on repolarization parameters and may have the potential to contribute to the development of repolarization abnormalities in individuals with pre-existing conditions. Due to the standardized and controlled study conditions and the isolated investigation of a specific physiological mechanism, the findings cannot be directly extrapolated to the complex real-life scenario of drowning.

## Introduction

Swimming and diving are popular recreational activities supporting an active lifestyle and improve wellbeing as well as reduce cardiovascular risk and prevent obesity (1, 2). In addition, learning to swim is essential to prevent drowning accidents especially in young people. Understanding the physiological responses to immersion and submersion as well as the mechanisms of drowning, is crucial for reducing drowning-related fatalities. Drowning is associated with the two events of immersion and submersion and, since it often occurs in water below body temperature, also with cooling (3).

At first immersion into cold water with the upper airways above water leads to the cold shock response due to skin cooling and activation of cutaneous cold receptors. This sympathetic response triggers tachycardia, reflex hyperventilation and impaired breath-holding and increases the risk of water aspiration. It begins at water temperatures below 25 °C, peaks at 10–15 °C, and lasts 2–3 min, with maximum intensity around 30 s (3–6). In adults, heart rate increases by 62.5% and respiratory rate by 450% within the first minute of cold water immersion (7). In children, the cold shock response is much less pronounced, leading to only a 26% increase in heart rate and a 55% increase in respiratory rate after 1 min (8). This age-related difference may be due to higher oxygen uptake, greater metabolic heat production or lower limb skin temperature in children (8). In our cohort, heart rate increased by 31% and respiratory rate by 58% within the first minute of immersion into 11 °C cold water (9). The cold shock response peaked at around 30 s, with acclimatization beginning by 60 s as heart and respiratory rates started to return to baseline (9). At second submersion, especially facial immersion and apnea trigger the parasympathetic diving response, resulting in bradycardia to conserve oxygen and extend underwater time (3). In adults, facial immersion reduces heart rate by 42% within 30 s (5). Our recent study in healthy children showed a heart rate decrease of 16% during dry apnea, 28% during face immersion, and 25% during full submersion within 30 s (10).

Simultaneous activation of the sympathetic cold shock response and the parasympathetic diving reflex during cold-water submersion can lead to autonomic conflict, which increases the risk of cardiac arrhythmias (3). Arrhythmia incidence in adults rises from 2% during cold-water immersion with free breathing to 82% when the face is submerged and breath is held (5). Reported events include atrial, nodal and ventricular ectopy as well as bradyarrhythmias (sinus arrest, AV block) and brief episodes of ventricular tachycardia such as torsade de pointes, often occurring during transitions in autonomic dominance, particularly at the end of breath-holding (5, 11, 12). Rapid autonomic shifts, such as facial immersion in cold water, may also be associated with delayed QT adaptation (QT hysteresis), which could contribute to ventricular arrhythmogenesis due to transient mismatch between heart rate and repolarization (5, 13–15). These arrhythmias may also contribute to potential cold water fatalities, as even non-fatal events can cause loss of consciousness and swimming incapacity, leading to secondary drowning. The risk of developing such arrhythmias is increased in individuals with pre-existing heart conditions, such as congenital heart defects or congenital conduction system disorders (3–5, 16).

Drowning is a leading cause of accidental death worldwide, particularly in young and non-swimmers. In fact, water immersion is a major cause of accidental death in both children and adults, regardless of whether the individuals actually drown (17, 18). Earlier interpretations of drowning based on lung water content or its absence (drowning vs. hypothermia or “dry drowning”) are now considered outdated, as most immersions are too brief to cause hypothermia. Current understanding highlights the cold shock response with impaired breathing and water aspiration as a key mechanism, even in experienced swimmers. In addition, autonomic conflict induced cardiac arrhythmias may contribute to sudden incapacitation and drowning but are often undetectable post-mortem, potentially mimicking drowning as the cause of death (5).

Most data on drowning physiology are derived from adult populations, although drowning represents a particular risk during childhood. There is currently almost no data on the drowning physiology of children, particularly on reactions to cold water immersion, with only two studies by Bird et al. (8, 19) and our recent study (9), while none addressing the effects of immersion on cardiac repolarization. Additionally, the data on swimming and diving in children with pre-existing heart conditions are currently scarce, while they are assumed to be at a particularly high risk. Due to limited experience and data on this topic, clear recommendations are lacking, often leading to restrictive patient counseling (20, 21). In this context, our research group carried out two pilot studies on immersion and submersion effects in young adults with congenital heart defects (22–24). Further research on the responses to immersion and submersion in children with congenital heart defects and congenital conduction system disorders requires reliable baseline data from healthy children. Therefore, we recently investigated the physiological diving response (10) and the adaptation to physical exertion during submersion and apnea (25). The cold shock response in healthy children has now been described by our research group (9). The current study aims to further investigate the effects of cold water immersion on repolarization patterns and arrhythmia burden. The key question is whether the cold shock reflex itself has arrhythmogenic potential even without facial immersion, and therefore without an autonomic conflict. It seeks to fill the existing data gap on swimming and diving physiology in healthy children and establish a structured data base for future studies involving children with cardiac preconditions, such as congenital heart defects or congenital conduction system disorders. Furthermore, it intends to enhance the knowledge on drowning mechanisms in children to prevent drowning accidents.

## Methods

### Participants

Healthy voluntary participants were recruited through local surveys and announcements in schools and swimming clubs in the weeks preceding the study. Potential participants and their guardians were approached using standardized, neutral study information. Participation was entirely voluntary, and both children and their guardians were explicitly informed that declining participation or withdrawing from the study at any time would not result in any disadvantages. Adequate time was provided to consider participation before enrollment. Written informed consent was obtained from all parents or legal guardians, and age-appropriate assent was obtained from the participating children.

Inclusion criteria were age of 8–14 years, ability to swim and overall health, especially no restrictions of cardiopulmonary function. Exclusion criteria were signs of reduced general condition and acute illness, signs of limitations of cardiopulmonary function, e.g., a newly diagnosed heart murmur, and intellectual disability or genetic disease. A relatively small sample size was deliberately chosen to allow an initial, controlled assessment of the effects in children, taking into account potential unknown parameter variability, with a larger follow-up study planned. The minimum age was set at 8 years to ensure that the children were old enough to understand the experiment, provide their consent, and participate voluntarily and cooperatively.

### Risk assessment and safety protocol

The study received ethical approval by the ethics committee of University of Leipzig and is listed under the reference 233/24-ek. The ethics committee raised no ethical or scientific concerns regarding the submitted study design and classified the investigation as involving low risk. The experimental design was carefully developed to minimize potential harm, with particular attention to the pediatric study population. Cold water immersion was performed under strictly controlled conditions in a safe environment. All tests were conducted in a small pool with a depth of 1 m, rather than in open water. The immersion time was limited to 1 min, allowing observation of the cold shock response while minimizing the risk of hypothermia. Anticipated risks were considered to be limited to transient increases in heart rate and respiratory rate, as well as potential changes in ECG parameters and the occurrence of mild arrhythmias, such as premature beats. As no facial immersion or apnea was involved, the risk of severe arrhythmias related to autonomic conflict was considered low. Furthermore, given the study population of healthy children, who can swim safely, the likelihood of adverse events was deemed minimal. Overall, the anticipated scientific and clinical value of the study was judged to outweigh the minimal and well-controlled risks associated with the intervention.

The tests were supervised by two medical doctors, including an experienced pediatric cardiologist and intensive care specialist, as well as a pediatric nurse and a study nurse. Continuous cardiorespiratory monitoring was maintained throughout the protocol. Emergency equipment and trained personnel were available on site at all times. Predefined termination criteria included completion of data acquisition, failure of monitoring equipment or the occurrence of clinically relevant arrhythmias or other adverse events. Adverse events were defined as any unexpected physiological or clinical response beyond transient, self-limiting changes such as short-term increases in heart rate or respiratory rate or mild tremor.

### Experimental protocol

Anthropometric data were measured. Data on potential medical history and medication were obtained from personal interviews. The testing took place in two pools of the first division soccer club RB Leipzig e.V. in the Red Bull stadium in Leipzig. The pools were the same size with a depth of 1 m and two steps, and were located right next to each other in the same room. Ambient temperature was around 25 °C, water temperature was 34 °C (close to thermoneutral) in the warm pool and 11 °C in the cold pool. A water temperature of 11 °C was selected, as previous studies in adults demonstrated the cold shock response to be most pronounced at 10–15 °C (4, 6). The lower bound of this range was chosen to elicit the strongest possible response, particularly since previous studies in children reported a less pronounced cold shock response compared to adults (8, 9). For comparison, a temperature of 34 °C was used, as it is close to thermoneutral and remains well above the threshold (∼25 °C) at which the cold shock response should be initiated (4, 6).

Prior to the start of the testing, all participants were instructed to the following protocol. The protocol consisted of two parts, one conducted in a warm pool, the other one in a cold pool. The participants wearing swimsuits or shorts quickly entered the warm water with the water reaching up to their necks. After the monitoring outside the pool, including drying and being wrapped in a towel, they were quickly lowered into the cold water in a seated position without moving themselves to avoid hesitation to fully enter the very cold water, again with the water reaching up to their necks. Immersion in both warm and cold water was completed within a maximum of 5 s for all participants.

Protocol:

1. 2 min of monitoring in a sitting position outside the pool
2. 1 min of immersion until the neck in warm (close to thermoneutral) water
3. 2 min of monitoring in a sitting position outside the pool
4. 1 min of immersion until the neck in cold water
5. 2 min of monitoring in a sitting position outside the pool

Skin temperature was measured continuously under water (on the participant's hand) as well as above water (on the participant's forehead) and ECG was conducted continuously by using a Dräger Infinity® 540 patient monitor (Dräger Medical GmbH, Lübeck, Germany). Additionally, a Lifecard CF® Holter-ECG-recorder was used to record heart rate and enable subsequent analysis of heart rate variability parameters and detection of arrhythmias (Spacelabs Healthcare GmbH, Nürnberg, Germany). Heart rate and respiratory rate were measured continuously using a Masimo Rad-97™ patient monitor with Masimo RD SET neo CS-3 sensors and RAS-45 rainbow acoustic sensors (Masimo Corporation, Irvine, USA).

For the arrhythmia detection, the ECGs were evaluated for arrhythmias and extrasystoles. The ECGs were then divided into respective phases (resting 1, warm water, resting 2, cold water, resting 3) based on the time indications. The times for atrioventricular conduction (PR interval), ventricular depolarization (QRS interval) and repolarization (QT interval) were measured four times per phase. Additionally, the QT interval was frequency-corrected with the RR interval according to Bazett's formula (QTc). Furthermore, the QT dispersion was calculated as a measure of regional differences in the duration of ventricular repolarization and therefore as an indirect sign of electrical inhomogeneity. The Tpeak-Tend (Tp-Te) interval was quantified as an indicator of the transmural gradient of repolarization, as well as Tp-Te dispersion. To illustrate the dynamics of the QTc interval and Tp-Te interval, the average interval of the first 30 s and the last 30 s, as well as the difference between them, were determined. The measurements were performed manually using lead II. The measurements were not averaged across multiple leads, but across four times per phase. All ECG measurements were performed by a single investigator to avoid interobserver variability. Intra- and interobserver variability were not assessed.

For heart rate variability analysis, the following parameters were used: standard deviation of NN intervals (SDNN), number of pairs of successive NNs that differ by more than 50 ms (sNN50), root mean square of successive differences (RMSSD) and triangular index, that describes the distribution of normal NN intervals. The larger these parameters are, the greater is heart rate variability, reflecting the balance between the sympathetic and parasympathetic nervous systems. Low values suggest tension and sympathetic activity, while high values indicate relaxation, parasympathetic activity or a healthy autonomic nervous system and a well-trained state. Nevertheless, heart rate variability parameters represent only indirect measures of autonomic regulation.

### Statistical analysis

For the statistical analysis, IBM SPSS Statistics for Mac (V29) was used. Non-parametric tests were used due to the small sample size and non-normal data distribution. Anthropometric data and rhythmological parameters were analyzed and gender-specific differences were compared by conducting a Mann–Whitney *U* test. ECG parameters, repolarization parameters and heart rate variability parameters were compared between warm and cold water by using a Wilcoxon signed rank test for dependent samples. The *p*-value is included in the manuscript. The significance level was set at *α* = 0.05.

## Results

In this study, 12 healthy children aged 9–13 years were enrolled, including 4 girls and 8 boys. All 12 participants finished the protocol including immersion into warm water (34 °C) and cold water (11 °C). Due to poor recording quality, which precluded reliable analysis and measurement of the ECGs, the analysis of the Holter-ECGs and thus the examination of arrhythmias and repolarization patterns was only possible for 9 participants, 2 girls and 7 boys. The 3 excluded participants consisted of 2 girls and 1 boy also aged 9–13 years, who did not differ in demographic or physiological characteristics from those included in the final analysis. No participant reported symptoms such as discomfort, dizziness, nausea, shortness of breath or heart palpitations. No adverse events were seen.

Table 1 shows the characteristics of the 9 participants including anthropometric and repolarization data. No significant differences between girls and boys could be observed. Given the unequal sex distribution, sex-specific comparisons should be interpreted with caution and are of an exploratory nature only.

**Table 1:** Characteristics of the study population.

Parameters	Total (*n* = 9) mean (min; max)	Female (*n* = 2; 22.2%) mean (min; max)	Male (*n* = 7; 77.8%) mean (min; max)	*p*-value
Age (years)	10.67 (9; 13)	9 (9; 9)	11 (9; 13)	0.111
Height (cm)	146.67 (134; 162)	146 (146; 146)	146.9 (134; 162)	0.889
Weight (kg)	35.19 (26.7; 46.8)	34.35 (34.2; 34.5)	35.4 (26.7; 46.8)	0.889
BMI (kg/m^2^)	16.29 (14.3; 19.7)	16.1 (16.0; 16.2)	16.3 (14.3; 19.7)	0.889
Mean PR (ms)	123.25 (110; 141.7)	112.81 (110; 115.6)	126.24 (110; 141.7)	0.222
Mean QRS (ms)	75.99 (68.5; 89.3)	73.33 (71.7; 75)	76.75 (68.5; 89.3)	0.5
Mean QT (ms)	297.33 (257.5; 317.5)	264.58 (257.5; 271.7)	306.68 (296.7; 317.5)	0.056
Mean QTc (ms)	382.22 (365.1; 421.1)	381.47 (379.2; 383.8)	382.43 (365.1; 421.1)	0.889
Mean Tp-Te (ms)	49.03 (40.75; 57)	49.06 (44.38; 53.75)	49.03 (40.75; 57)	1.000

### Arrhythmias

One proband showed 5 premature ventricular contractions (PVC) and 1 premature atrial contraction (PAC) in warm water as well as 7 PVC in cold water and 9 PVC during monitoring outside the pool, without reporting any symptoms. Another proband showed 4 PVC during monitoring outside the pool but none in the water, also without reporting any symptoms. There was no pathological prolongation or shortening of PR interval, QRS interval or QTc interval. One participant exhibited a high-normal QTc interval with a maximum of 462 ms. As shown in Table 2 there are no significant differences in the rhythmological parameters between warm and cold water.

**Table 2:** Rhythmological parameters in warm and cold water.

Parameters	Warm water mean (min; max)	Cold water mean (min; max)	*p*-value
Mean PR (ms)	125 (110; 145)	120.56 (105; 142.5)	0.260
Mean QRS (ms)	74.44 (62.5; 90)	77.22 (70; 85)	0.102
Mean QT (ms)	297.22 (245; 337.5)	293.24 (265; 317.5)	0.953

### Repolarization patterns

**Figure 1:**
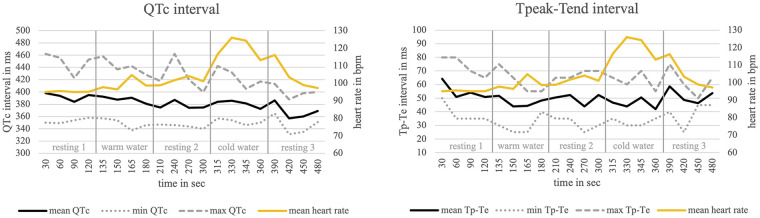
QTc interval, Tpeak-Tend (Tp-Te) interval and mean heart rate over time during immersion in warm and cold water, as well as the surrounding resting periods.

Figure 1 shows the progression of the frequency-corrected QT (QTc) interval, Tpeak-Tend (Tp-Te) interval and the mean heart rate over time during immersion in warm and cold water, as well as the surrounding resting periods. As shown in Table 3, no significant differences were found in the parameters of repolarization between warm and cold water. When comparing baseline (resting 1) with warm water, no significant differences were observed in the repolarization parameters (Table 4). However, when comparing baseline with cold water, significant differences were found in the parameters mean Tp-Te and maximum Tp-Te, with both values being significantly shorter during cold water immersion than in air. Mean Tp-Te showed a decrease of 16.6%, while maximum Tp-Te decreased by 10.8% (Table 5).

**Table 3:** Comparison of the repolarization parameters between warm and cold water.

Parameters	Warm water mean (min; max)	Cold water mean (min; max)	*p*-value
Mean QTc (ms)	387.89 (361.5; 428.3)	381.39 (359; 423.3)	0.441
Min QTc (ms)	365.11 (337; 397)	366 (346; 402)	0.953
Max QTc (ms)	412.33 (370; 358)	397.78 (365; 442)	0.183
QTc first 30 s (ms)	389.89 (355.5; 444)	381 (348.5; 440)	0.575
QTc last 30 s (ms)	385.89 (353; 429.5)	374.33 (342; 393.5)	0.236
Difference in QTc between first and last 30 s (ms)	−4 (−68; 35)	−6.67 (−47.5; 23)	0.515
QT dispersion (ms)	22.22 (10; 40)	30 (10; 50)	0.250
Mean Tp-Te (ms)	47.01 (37.5; 60)	45.88 (36.25; 52.5)	0.859
Min Tp-Te (ms)	36.67 (25; 50)	35.56 (30; 40)	0.942
Max Tp-Te (ms)	58.33 (45; 75)	57.22 (40; 70)	0.516
Tp-Te dispersion (ms)	21.67 (5; 40)	21.67 (10; 35)	0.852
Tp-Te first 30 s (ms)	47.78 (27.5; 70)	45.28 (35; 55)	0.677
Tp-Te last 30 s (ms)	46.39 (32.5; 52.5)	46.67 (32.5; 52.5)	0.750
Difference in Tp-Te between first and last 30 s (ms)	−1.39 (−25; 20)	1.39 (−22.5; 12.5)	0.553

**Table 4:** Comparison of the repolarization parameters between baseline and warm water.

Parameters	Baseline mean (min; max)	Warm water mean (min; max)	*p*-value
Mean QTc (ms)	392.75 (352.8; 448.5)	387.89 (361.5; 428.3)	0.345
Min QTc (ms)	379.67 (349; 423)	365.11 (337; 397)	0.116
Max QTc (ms)	406.33 (358; 462)	412.33 (370; 358)	0.917
QT dispersion (ms)	25.00 (10; 40)	22.22 (10; 40)	0.334
Mean Tp-Te (ms)	55 (41.3; 72.5)	47.01 (37.5; 60)	0.140
Min Tp-Te (ms)	45.83 (35; 60)	36.67 (25; 50)	0.334
Max Tp-Te (ms)	64.17 (50; 80)	58.33 (45; 75)	0.071
Tp-Te dispersion (ms)	18.33 (10; 30)	21.67 (5; 40)	0.748

**Table 5:** Comparison of the repolarization parameters between baseline and cold water.

Parameters	Baseline mean (min; max)	Cold water mean (min; max)	*p*-value
Mean QTc (ms)	392.75 (352.8; 448.5)	381.39 (359; 423.3)	0.249
Min QTc (ms)	379.67 (349; 423)	366 (346; 402)	0.115
Max QTc (ms)	406.33 (358; 462)	397.78 (365; 442)	0.345
QT dispersion (ms)	25.00 (10; 40)	30 (10; 50)	0.680
Mean Tp-Te (ms)	55 (41.3; 72.5)	45.88 (36.25; 52.5)	**0** **.** **046** *
Min Tp-Te (ms)	45.83 (35; 60)	35.56 (30; 40)	0.072
Max Tp-Te (ms)	64.17 (50; 80)	57.22 (40; 70)	**0** **.** **041** *
Tp-Te dispersion (ms)	18.33 (10; 30)	21.67 (10; 35)	0.102

* Significant at a level of *α* = 0.05.

Bold values marked with a * indicate statistically significant differences.

### Heart rate variability

Table 6 shows the heart rate variability parameters including SDNN, sNN50, RMSSD and triangular index in warm and cold water, including significant differences.

**Table 6:** Heart rate variability parameters in warm and cold water.

Parameters	Warm water mean (min; max)	Cold water mean (min; max)	*p*-value
SDNN (ms)	77,89 (38; 107)	57,60 (19; 131)	**0** **.** **021** *
sNN50	36,22 (12; 83)	29,50 (2; 129)	0.109
RMSSD	47,56 (26; 93)	41,10 (17; 65)	0.154
Triangular index	11,67 (4; 19)	7,50 (4; 11)	**0** **.** **037** *

* Significant at a level of *α* = 0.05.

Bold values marked with a * indicate statistically significant differences.

## Discussion

The present study provides first-real life data on the influence of cold shock response on changes in repolarization patterns and the development of arrhythmias during immersion of healthy children into 11 °C cold water. Significant differences in skin temperature, heart rate and respiratory rate were reported between warm water and cold water immersion (9). In warm water, heart and respiratory rates remained relatively constant, whereas in cold water, with a significant drop in skin temperature by 8 °C, heart rate increased by 31% and respiratory rate increased by 58% (9). Regarding temperature assessment, it should be noted that only skin temperature was measured at two different sites: on the back of the hand (underwater) and on the forehead (above water). As only peripheral skin temperature was recorded, it can be assumed that these values were already lower than central skin temperature at baseline. In addition, the children were already slightly peripherally cooled during the monitoring periods prior to water immersion (9). Furthermore, the absence of core temperature measurement represents a methodological limitation of the study, although the short immersion duration likely minimized the risk of clinically relevant hypothermia. Skin temperature measured under water primarily reflects the direct cooling effect of water exposure rather than intrinsic thermoregulatory processes.

The analysis of heart rate variability clearly shows that immersion into cold water is a strong stressor and triggers a significant sympathetic response. All heart rate variability parameters are lower in cold water, with some being significantly reduced, indicating decreased heart rate variability and therefore increased sympathetic activity. It should be noted, however, that heart rate variability parameters provide only indirect insight into autonomic regulation.

The QT interval represents the duration of ventricular repolarization. It is dependent on heart rate and can be corrected for heart rate as QTc. In children aged 9–13 years, a QTc interval is considered physiological if it is below 460 ms in girls and below 440 ms (or below 450 ms during mild exertion) in boys and above 350 ms (26, 27). A prolonged QTc interval indicates a repolarization disorder and may promote the development of ventricular arrhythmias, particularly torsade de pointes tachycardia. In our cohort, the average QTc was 382 ms, with a slightly, but not significantly shorter QTc duration in cold water compared to warm water and compared to baseline. The difference in QT duration between warm and cold water was also not significant, although the QT interval was slightly lower in cold water. Normally, sympathetic activation and the resulting tachycardia would be expected to shorten the QT interval. However, despite a significant 31% increase in heart rate in cold water, this expected QT shortening does not occur. Thus, we were able to identify an indication of QT hysteresis during cold water immersion even without facial immersion. Nevertheless, the QTc interval also remains nearly unchanged. Therefore, the present findings rather suggest QT hysteresis but do not allow for a definitive demonstration of it. The simultaneous occurrence of tachycardia and a prolonged, improperly adjusted QT interval could promote an increasing risk of ventricular tachycardias. Since there are no significant differences in QT and QTc between warm and cold water, QT hysteresis and thus the risk of arrhythmia development is likely to be minimal in these healthy children. However, this effect could be much more pronounced in children with preexisting heart conditions.

QT dispersion, defined as the difference between the longest and shortest QT intervals, serves as an indicator for the spatial inhomogeneity of ventricular repolarization. It is assumed that an increasing time difference in the repolarization process across different myocardial regions facilitates the occurrence of reentry phenomena and, consequently, the potential development of ventricular tachyarrhythmias. For this reason, QT dispersion is considered a risk marker for sudden cardiac death by some authors. Due to the lack of standardized methods for determining QT dispersion, as well as the absence of reference and pathological threshold values, its measurement is typically avoided in clinical practice. However, it can be assumed that a QT dispersion of <65 ms indicates a physiological QT dispersion in adults. A pathological QT dispersion >65 ms is commonly observed in patients with structural heart diseases or malignant ventricular tachyarrhythmias (28). QT dispersion in our subjects ranged between 10 and 50 ms, with slightly but not significantly higher values in cold water compared to warm water. The QTc dispersion can also be calculated. However, it is recommended not to correct the QT dispersion for heart rate in children, as the QTc dispersion is influenced by the commonly occurring sinus arrhythmia in childhood (29). Therefore, the authors also refrain from mentioning the QTc dispersion here.

The Tpeak-Tend (Tp-Te) interval, defined as the interval from peak to end of the T wave, represents the transmural gradient of repolarization. It is considered a reliable marker for predicting ventricular tachycardia, ventricular fibrillation and sudden cardiac death and is frequently used for risk stratification in patient groups with various cardiac conditions, such as congenital arrhythmias, ischemic artery disease, heart failure or hypertension. An increased variation in repolarization between the heart's base and apex, either intramurally or within the interventricular septum, contributes to a higher risk of ventricular arrhythmias, particularly in individuals with channelopathies (30). In healthy children, the Tp-Te interval averages around 60 ms, with a range of 20–120 ms, while boys and older children tend to have longer intervals. The Tp-Te interval prolongs as heart rate decreases (31). Similar to QT dispersion, Tp-Te dispersion can be calculated as the difference between the longest and shortest Tp-Te interval. In healthy children, it averages approximately 40 ms, with a range of 6–80 ms, while Tp-Te dispersion increases with age but does not show differences between boys and girls (31). The Tp-Te interval in our cohort averaged 49 ms and was slightly lower in cold water than in warm water but significantly lower than the baseline Tp-Te in air. Tp-Te dispersion showed the same mean value in both warm and cold water and was slightly higher than at baseline. This demonstrates the typical shortening of the Tp-Te interval with increasing heart rate (31) as well as a noticeable influence of the cold shock response on the transmural gradient of repolarization. However, this gradient is lower than in air, and thus the actual risk of arrhythmia is likely reduced. At this point, it can only be speculated very carefully that in patients with congenital repolarization disorders, the situation may differ and the arrhythmia risk could increase. Due to the absence of clinically relevant arrhythmias in this healthy study population, and given that a shortening of the Tp–Te interval is generally not considered a classical proarrhythmic finding, no hypotheses regarding arrhythmia risk in vulnerable patient populations can be derived from the direct observations in this cohort. The findings should be interpreted with caution, while future studies will need to address this issue.

Although there is an indication of a missing or insufficient adaptation of the QT interval to the suddenly increased heart rate (QT hysteresis) during cold water immersion, no significant differences in repolarization patterns between warm and cold water could be demonstrated. However, during cold water immersion, a significantly shorter Tp-Te interval was observed compared to the baseline value in air, suggesting a notable impact on the transmural gradient of repolarization. Two children exhibited premature ventricular and atrial contractions, which occurred independently of immersion and were not associated with any symptoms. Premature beats are common in children and adolescents and are usually of no pathological relevance. Nevertheless, in the context of drowning, they may potentially act as triggers for malignant arrhythmias, however, this remains purely hypothetical in the present setting. Despite these premature contractions, no cardiac arrhythmias were observed during cold water immersion. This may be explained by the absence of facial immersion, as the risk of arrhythmias significantly increases during simultaneous cold-water and facial immersion with apnea, as a result of an autonomic conflict. Nevertheless, the cold shock response itself also appears to have some influence on repolarization parameters and may have the potential to contribute to the development of repolarization abnormalities, particularly in predisposed individuals or patients with pre-existing conditions.

Due to the very small sample size, the short exposure duration and the absence of clinically relevant arrhythmias, the present study should be considered exploratory and its findings should be regarded as hypothesis-generating. Conclusions regarding the arrhythmogenic potential of the cold shock response in healthy children should therefore be interpreted with caution. As no children with pre-existing cardiac conditions were included in the study population, the impact of the cold shock response on cardiac repolarization and the development of arrhythmias in this group can only be discussed hypothetically. Further studies including larger cohorts of both healthy children and those with underlying cardiac conditions are required to confirm these findings and to allow for more robust conclusions.

Real-life drowning represents a complex and multifactorial process involving hypoxia, psychological stress, thermal stress and the concurrent activation of the sympathetic cold shock response and the parasympathetic diving reflex. This interaction is considered central to the arrhythmogenic potential observed in drowning scenarios. In contrast, the present study was specifically designed to investigate the isolated cold shock response under standardized and controlled conditions. By deliberately excluding facial immersion and apnea, key components of the drowning process were not elicited, thereby limiting the physiological complexity of the model. Moreover, important factors inherent to real-life drowning, such as panic, uncontrolled breathing, water aspiration, prolonged exposure and variable environmental conditions, were not replicated. Consequently, the findings are not generalizable, cannot be directly extrapolated to real-world drowning scenarios and should be interpreted with caution. Importantly, this reductionist approach reflects not only a methodological decision but also an ethical necessity. Experimental investigation of drowning physiology in humans, particularly in pediatric populations, cannot be conducted under conditions that would reproduce the full pathophysiological cascade due to unacceptable risks. Therefore, the study of drowning-related mechanisms must rely on controlled, standardized models focusing on individual physiological components. Within this context, the present findings provide valuable, hypothesis-generating insights into the isolated effect of cold shock response on repolarization patterns and the development of arrhythmias in healthy children, while acknowledging the inherent limitations in generalizability.

## Conclusion

The present study is the first providing exploratory data on repolarization patterns and arrhythmia burden in healthy children during the immersion into 11 °C (52°F) cold water. Data suggest that cold water immersion acts as a potent sympathetic stressor and may be associated with a mild QT hysteresis. Compared to baseline values in air, a significantly shorter Tp-Te interval was observed in cold water, indicating a significant influence on the transmural gradient of repolarization. Although no arrhythmias were detected during cold water immersion in healthy children, the cold shock response itself appears to have some influence on repolarization parameters. Due to the standardized and controlled study conditions and the isolated investigation of a specific physiological mechanism, the findings cannot be directly extrapolated to the complex real-life scenario of drowning.

## Limitations

The current study is limited by the small number of participants, the narrow age range of the participants and the male dominance in the gender ratio, therefore a bias due to proband selection is possible. The small number of patients resulted from very restrictive ethical approval for a potentially harmful test protocol in children. With a small sample size and non-normally distributed data, the statistical power is limited. Due to the very small sample size, the short exposure duration and the absence of clinically relevant arrhythmias, the present study should be considered exploratory and its findings should be regarded as hypothesis-generating.

The exclusive measurement of peripheral skin temperature represents a limitation of the study, as it was likely lower than central skin temperature due to peripheral cooling of the participants during the monitoring period. Moreover, the lack of core temperature assessment constitutes a methodological limitation, although the brief duration of immersion likely reduced the risk of clinically significant hypothermia.

The analysis of the Holter ECGs and the measurement of the corresponding intervals by a single investigator also represent a limitation of the study. Intra- and interobserver variability were not assessed.

Furthermore, the measurements were conducted using materials and devices not qualified for use underwater. Although the devices were well-adapted to be waterproof, we cannot completely exclude aberrant values because of off-label use.

Due to the standardized and controlled study conditions and the isolated investigation of a specific physiological mechanism, the findings cannot be directly extrapolated to the complex real-life scenario of drowning.

## Data Availability

The raw data supporting the conclusions of this article will be made available by the authors, without undue reservation.
